# Supplementary material for: Discovery and application of insertion-deletion (INDEL) polymorphisms for QTL mapping of early life-history traits in Atlantic salmon
Source: BMC Genomics. 2010 Mar 8;11:156. doi: 10.1186/1471-2164-11-156 (PMC2838853; doi:10.1186/1471-2164-11-156)
Supplement: Additional file 2 — Information on developed 76 locus single-run INDEL panel in Atlantic salmon. Information on fluorescence labeling, primer concentrations, PCR pooling and links to alignments, INDEL motifs and GENESCAN (Burge and Karlin 1997) predictions of genes/exons are available in html format. [file 1471-2164-11-156-S2.ZIP › Additionalfile2/snpsummary548.html]

```
Cluster 59 Contig 1

prev  Summary    Contig List  next
```

Size of Consensus sequence = 1581

Number of sequences = 33

Minimum redundancy = 6

Key

A gi|84204196|gb|DW177706.1|DW177706 SGP304314 Atlantic salmon Brain II cDNA library Salmo salar cDNA clone SS0084E05 5', mRNA sequence  
B gi|117540952|gb|EG872397.1|EG872397 EST\_ssal\_eve\_18563 ssaleve thyroid Salmo salar cDNA Salmo salar cDNA clone ssal\_eve\_525\_113\_fwd 3', mRNA sequence  
C gi|117423751|gb|EG755975.1|EG755975 EST\_ssal\_sjb\_3774 ssalsjb mixed\_tissue Salmo salar cDNA Salmo salar cDNA clone ssal\_sjb\_010\_182\_fwd 3', mRNA sequence  
D gi|29324162|gb|CB512936.1|CB512936 ssalrgb544211\_rev mixed\_tissue Salmo salar cDNA, mRNA sequence  
E gi|117853293|gb|EG925989.1|EG925989 EST\_ssal\_evf\_28547 ssalevf mixed\_tissue Salmo salar cDNA Salmo salar cDNA clone ssal\_evf\_537\_326\_fwd 3', mRNA sequence  
F gi|117435325|gb|EG767548.1|EG767548 EST\_ssal\_evd\_43576 ssalevd thymus Salmo salar cDNA Salmo salar cDNA clone ssal\_evd\_558\_184\_fwd 3', mRNA sequence  
G gi|84568016|gb|DW339635.1|DW339635 SGP302396 Atlantic salmon Ovaries cDNA library Salmo salar cDNA clone KG4-2695 5', mRNA sequence  
H gi|117448619|gb|EG780838.1|EG780838 EST\_ssal\_evd\_37538 ssalevd thymus Salmo salar cDNA Salmo salar cDNA clone ssal\_evd\_550\_047\_fwd 3', mRNA sequence  
I gi|84568074|gb|DW339693.1|DW339693 SGP302455 Atlantic salmon Ovaries cDNA library Salmo salar cDNA clone KG4-2765 5', mRNA sequence  
J gi|117451228|gb|EG783447.1|EG783447 EST\_ssal\_evd\_39886 ssalevd thymus Salmo salar cDNA Salmo salar cDNA clone ssal\_evd\_553\_151\_fwd 3', mRNA sequence  
K gi|117478288|gb|EG810507.1|EG810507 EST\_ssal\_evd\_30776 ssalevd thymus Salmo salar cDNA Salmo salar cDNA clone ssal\_evd\_540\_223\_fwd 3', mRNA sequence  
L gi|117513426|gb|EG845185.1|EG845185 EST\_ssal\_eve\_10015 ssaleve thyroid Salmo salar cDNA Salmo salar cDNA clone ssal\_eve\_513\_215\_fwd 3', mRNA sequence  
M gi|117853292|gb|EG925988.1|EG925988 EST\_ssal\_evf\_28546 ssalevf mixed\_tissue Salmo salar cDNA Salmo salar cDNA clone ssal\_evf\_537\_326\_rev 5', mRNA sequence  
N gi|24385946|gb|CA055703.1|CA055703 ssalrgb511378 mixed\_tissue Salmo salar cDNA, mRNA sequence  
O gi|24389043|gb|CA058800.1|CA058800 ssalrgb544211 mixed\_tissue Salmo salar cDNA, mRNA sequence  
P gi|117509821|gb|EG841580.1|EG841580 EST\_ssal\_eve\_4316 ssaleve thyroid Salmo salar cDNA Salmo salar cDNA clone ssal\_eve\_504\_333\_fwd 3', mRNA sequence  
Q gi|117509822|gb|EG841581.1|EG841581 EST\_ssal\_eve\_4317 ssaleve thyroid Salmo salar cDNA Salmo salar cDNA clone ssal\_eve\_504\_333\_rev 5', mRNA sequence  
R gi|117513415|gb|EG845174.1|EG845174 EST\_ssal\_eve\_10014 ssaleve thyroid Salmo salar cDNA Salmo salar cDNA clone ssal\_eve\_513\_215\_rev 5', mRNA sequence  
S gi|117470492|gb|EG802711.1|EG802711 EST\_ssal\_evd\_59223 ssalevd thymus Salmo salar cDNA Salmo salar cDNA clone ssal\_evd\_579\_361\_fwd 3', mRNA sequence  
T gi|117540941|gb|EG872386.1|EG872386 EST\_ssal\_eve\_18562 ssaleve thyroid Salmo salar cDNA Salmo salar cDNA clone ssal\_eve\_525\_113\_rev 5', mRNA sequence  
U gi|117486888|gb|EG819105.1|EG819105 EST\_ssal\_evd\_21175 ssalevd thymus Salmo salar cDNA Salmo salar cDNA clone ssal\_evd\_527\_131\_rev 5', mRNA sequence  
V gi|24348739|gb|CA044778.1|CA044778 ssalmgf003047 gut Salmo salar cDNA, mRNA sequence  
W gi|117840837|gb|EG913533.1|EG913533 EST\_ssal\_evf\_15337 ssalevf mixed\_tissue Salmo salar cDNA Salmo salar cDNA clone ssal\_evf\_519\_013\_rev 5', mRNA sequence  
X gi|117470494|gb|EG802713.1|EG802713 EST\_ssal\_evd\_59224 ssalevd thymus Salmo salar cDNA Salmo salar cDNA clone ssal\_evd\_579\_361\_rev 5', mRNA sequence  
Y gi|117435326|gb|EG767549.1|EG767549 EST\_ssal\_evd\_43577 ssalevd thymus Salmo salar cDNA Salmo salar cDNA clone ssal\_evd\_558\_184\_rev 5', mRNA sequence  
Z gi|117448618|gb|EG780837.1|EG780837 EST\_ssal\_evd\_37537 ssalevd thymus Salmo salar cDNA Salmo salar cDNA clone ssal\_evd\_550\_047\_rev 5', mRNA sequence  
a gi|117850153|gb|EG922849.1|EG922849 EST\_ssal\_evf\_1920 ssalevf mixed\_tissue Salmo salar cDNA Salmo salar cDNA clone ssal\_evf\_006\_287\_fwd 3', mRNA sequence  
b gi|117451227|gb|EG783446.1|EG783446 EST\_ssal\_evd\_39885 ssalevd thymus Salmo salar cDNA Salmo salar cDNA clone ssal\_evd\_553\_151\_rev 5', mRNA sequence  
c gi|24351349|gb|CA045179.1|CA045179 ssalema001022 gut Salmo salar cDNA, mRNA sequence  
d gi|117498854|gb|EG831071.1|EG831071 EST\_ssal\_eve\_43256 ssaleve thyroid Salmo salar cDNA Salmo salar cDNA clone ssal\_eve\_558\_314\_rev 5', mRNA sequence  
e gi|117861007|gb|EG933703.1|EG933703 EST\_ssal\_evf\_35491 ssalevf mixed\_tissue Salmo salar cDNA Salmo salar cDNA clone ssal\_evf\_547\_093\_fwd 3', mRNA sequence  
f gi|24381614|gb|CA051371.1|CA051371 ssalrgb526378 mixed\_tissue Salmo salar cDNA, mRNA sequence  
g gi|117861006|gb|EG933702.1|EG933702 EST\_ssal\_evf\_35490 ssalevf mixed\_tissue Salmo salar cDNA Salmo salar cDNA clone ssal\_evf\_547\_093\_rev 5', mRNA sequence

4 SNPs detected

A B C D E F G H I J K L M N O P Q R S T U V W X Y Z a b c d e f g  cosegregation weighted

888 . . . . . . . T G T G T G G G G G T T . G G G T G . G T G G G . .   3/4 50.00
1099 . . . . . . . . C C G C G G G G G C C C C C G C G . G C G G G G G   1/4 18.18
1200 . . . . . . . . . - C - C C C C C - - - C C C - C . C - C C C C C   3/4 52.27
1201 . . . . . . . . . - A - A A A A A - - - A A A - A . A - A A A A A   3/4 52.27
